# Supplementary material for: The key role of sufficiency for low demand-based carbon neutrality and energy security across Europe
Source: Nat Commun. 2024 Oct 19;15:9043. doi: 10.1038/s41467-024-53393-0 (PMC11490562; doi:10.1038/s41467-024-53393-0)
Supplement: Supplementary file 1 — Supplementary Information [file 41467_2024_53393_MOESM1_ESM.pdf]

Supplementary Information for the article:

The key role of sufficiency for low demand-based carbon neutrality and energy security across Europe

Frauke Wiese[1 * a], Nicolas Taillard[2 a], Emile Balembois[2], Benjamin Best[3], Stephane Bourgeois[2] José Campos[4] Luisa Cordroch[1] Mathilde Djelali[2] Alexandre Gabert[2] Adrien Jacob[2] Elliott Johnson[5] Sébastien Meyer[6,7] Béla Munkácsy[4] Lorenzo Pagliano[8] Sylvain Quoilin[9] Andrea Roscetti[8,10] Johannes Thema[11,1] Paolo Thiran[7] Adrien Toledano[2] Bendix Vogel[1,12] Carina Zell-Ziegler[13,14] Yves Marignac[2]

1 Department of Sustainable Energy Transition, Europa-Universität Flensburg, Flensburg, Germany

2 négaWatt Association, BP 16280 Alixan, 26958 VALENCE Cedex 9, France

3 City of Bonn, Climate Neutral Bonn 2035 Program Office, Bonn, Germany

4 Department of Environmental and Landscape Geography, ELTE University, Budapest, Hungary

5 Sustainability Research Institute, University of Leeds, Leeds, UK

6 negaWatt Belgium, Rue du Blanc-Ry 163, Ottignies-Louvain-la-Neuve 1342, Belgium

7 Institute of Mechanics, Materials and Civil engineering, Université Catholique de Louvain, Louvain-la-Neuve, Belgium

8 Architecture and Urban Studies Department, Politecnico di Milano, Italy

9 Integrated and Sustainable Energy Systems, University of Liege, Belgium

10 Università della Svizzera italiana, Accademia di Architettura, Switzerland

11 Energy, Transport and Climate Policy Division, Wuppertal Institute for Climate, Environment and Energy, Wuppertal, Germany

12 Potsdam Institute for Climate Impact Research (PIK) e.V., Potsdam, Germany

13 Department of Landscape Planning and Development, Technische Universität Berlin, Berlin, Germany

14 Energy & Climate Division, Oeko-Institut, Berlin, Germany

Corresponding author[*]: Frauke Wiese frauke.wiese@uni-flensburg.de

[a] both authors have equally contributed to the work of this paper

This files contains the following supplementary information:

- Supplementary Method: Bioenergy potential applied in the CLEVER scenario
- Supplementary Discussion: Planetary boundary aspects in the CLEVER scenario
- Supplementary Note: Main Policy Strategies to achieve energy sufficiency in the CLEVER scenario by sector
- Supplementary Table 1-7
  - Supplementary Table 1: Historic and scenario data of average daily diet of an EU28 citizen
  - Supplementary Table 2: Consideration of the use of biogas resources in CLEVER
  - Supplementary Table 3: Consideration of the use of solid bioenergy in CLEVER
  - Supplementary Table 4: Consideration of the use of biofuels in CLEVER
  - Supplementary Table 5: Comparison of the bioenergy potential in different studies and scenarios
  - Supplementary Table 6: Overview sufficiency policy strategies in the CLEVER scenario
  - Supplementary Table 7: Overview countries in the CLEVER scenario (EU30)
- Supplementary Figure 1-5
  - Supplementary Figure 1: Schema of the systemic land use modelling tool ensuring consistency of land uses and supply balances for a given input
  - Supplementary Figure 2: Resulting evolution of land-use in EU28 based on the assumed measures in the CLEVER scenario
  - Supplementary Figure 3: Evolution of European imports/exports
  - Supplementary Figure 4: Overall change in bioenergy production
  - Supplementary Figure 5: Types of instruments for sufficiency applied in the CLEVER scenario by sector
- Supplementary References

Supplementary Method: Bioenergy potential applied in the CLEVER scenario

This supplementary material summarises the main aspects and resulting numbers on the modelling of the bioenergy potential for the CLEVER scenario.

Modelling approach

The model applied, MoSUT[1] [p.25], represents the agricultural and food system. Essential properties of the model:

- Physical: describing mass flows (mass of matter, food, feed, nitrogen, protein, gas, water, fuels), energy flows and surfaces used; analysing the supply and consumption balance of main agricultural and food products
- Normative: no optimisation by prices/production costs; focus on social and economic impacts of the trajectory
- Recursive / Back-forecasting: targeting several issues at the same time, gradually adjusting available levers; focus on trade-offs of the targets.

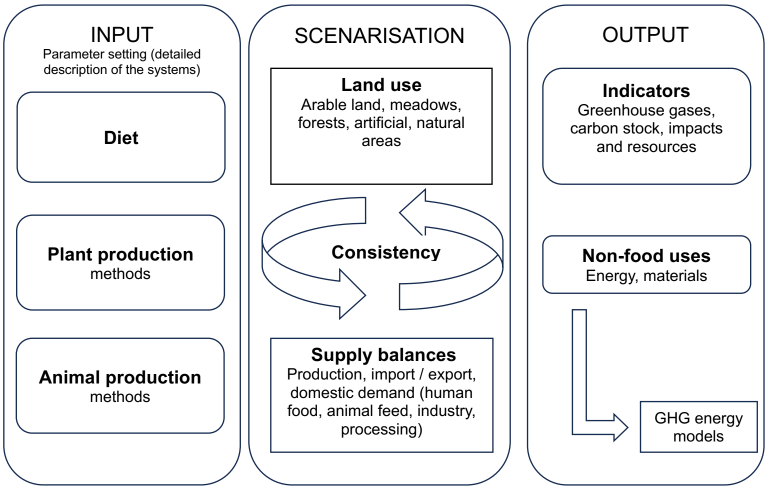

Supplementary Figure 1: Schema of the systemic land use modelling tool MoSUT (Modèle systémique d'utilisation des terres: Systemic model of land use)[2]. The bottom-up physical model ensures consistency of land uses and supply balances for a given input. For the CLEVER (Collaborative Low Energy Vision for the European Region) scenario it enables a consistent scenario regarding the food/energy/climate/land-use nexus. The core is a supply and consumption balance without cost-optimisation. 22 main crops, which are 90% of agricultural land area in Europe are modelled in detail based on EUROSTAT (statistical office of the European Union) and FAOSTAT (statistics of the Food and Agriculture Organization of the United Nations) data while for further 100 crops aggregated information is included.

Demand side

Demand is mainly determined by food demand for an assumed average diet, demographic trends and changes in food waste. Based on that, food supply is reduced by 11% expressed on a protein basis and by 6% on a calorific basis.

An increase by 12% of physical need is assumed due to an upward trend in average height and body mass index, and then a peak and then a decrease is assumed due to the success of public health policies, which also includes lifestyle changes and is partly related to the energy transition. For example, an increase in active modes in transport and healthier diets.

Diet has been modelled based on the historic FAO Food Balance[3] as well as key recommendations[4], such as shifting to a more plant-based diet and reducing over-consumption. The modelling has been done per country to be able to take national contexts and its effect on dietary habits into account. Supplementary Table 1 shows results for an average European in 2050, compared with historical data.

Supplementary Table 1: Historic and scenario data of average daily diet of an EU28 citizen, based on FAO (Food and Agriculture Organization of the United Nations) Food balance[3]. Unit: Gram (g) per day on average

	2014-2018 consumption (g/day)	2050 consumption (g/day)
Cereals - Excluding Beer	357	394
Starchy Roots	178	129
Sugar & Sweeteners	114	81
Pulses	7	20
Treenuts	10	16
Oilcrops	10	12
Vegetable Oils	45	74
Vegetable	294	314
Fruits - Excluding Wine	233	360
Stimulants	14	14
Spices	2	2
Alcoholic Beverages	269	173
Meat	218	133
Offals	7	8
Milk - Excluding Butter + Animal fats	736	581
Eggs	33	30
Fish, Seafood	63	52

It is assumed that food waste across the supply chain is reduced by half.

Supply side

Land-use change

Influence factors for land-use changes in this scenario are

- Limited urban sprawl: net increase of artificial areas of 3Mha
- Re-naturing policies, ecological restoration, land abandonment trends, spontaneous afforestation of former fallow land, afforestation policies on arable land, policies to conserve and protect permanent meadows and grassland:
  - Increase of natural and semi-natural areas: 2 Mha
  - Increase of forests: 3 Mha
  - Loss of grassland/meadows to 1 Mha to the benefit of moorland and new forest areas
- Sharp reduction of fodder crops on arable land, slight decrease of grain crops, increase in vegetable and fruit crops:
  - Decrease of agricultural area: 174 to 167 Mha

The resulting distribution of land-use and its changes from 2010 to 2050 are shown in Supplementary Figure 2.

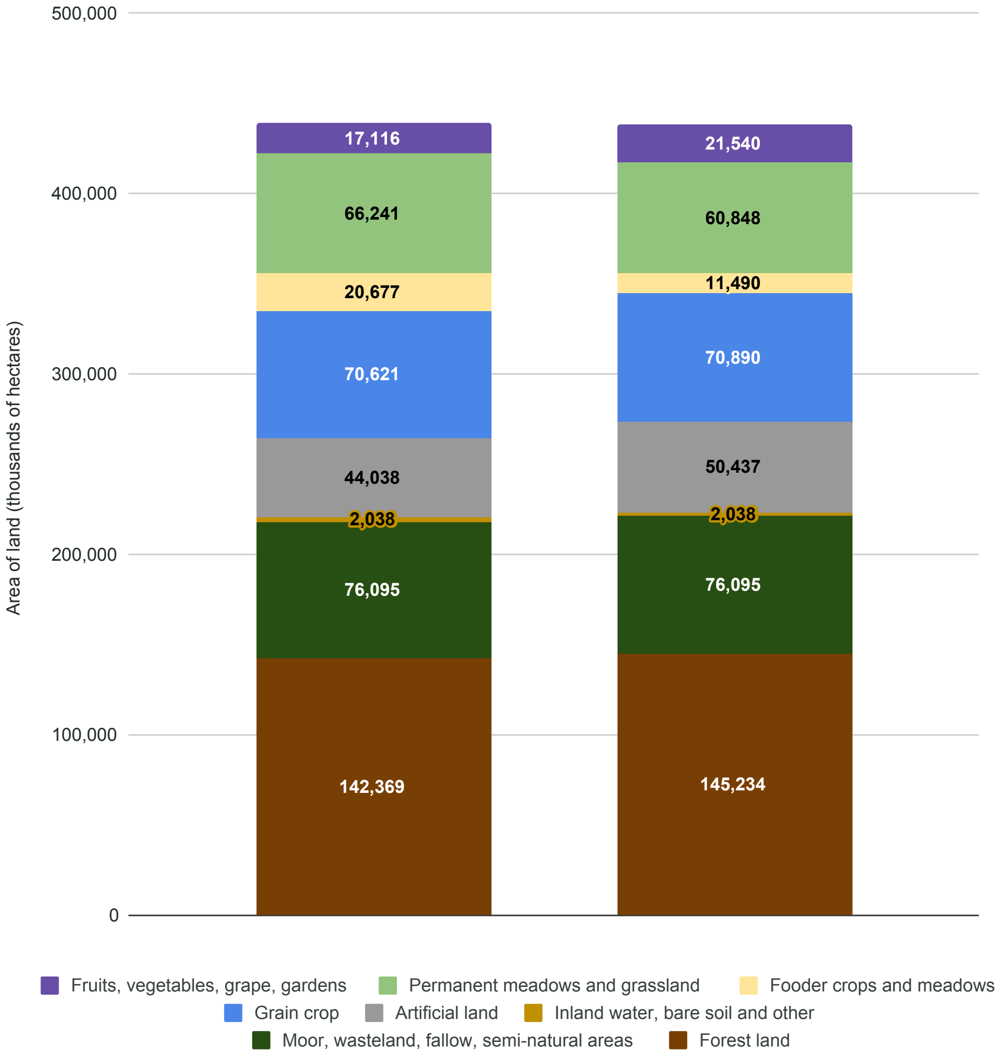

Supplementary Figure 2: Resulting evolution of land-use in EU28 based on the assumed measures in the CLEVER (Collaborative Low Energy Vision for the European Region) scenario (Unit: 1000 hectares). Data and figure are based on Couturier et al. (2022)[2]. The biggest changes are a reduction of land used for fooder crops and meadows as well as permanent meadows and grassland until 2050 compared to 2010 on the one side and an increase of forest and land for fruit, vegetable, grape and gardens on the other side.

Agroecology

Agroecology[5] is the agricultural practice most used in the CLEVER pathway. It is based on 6 principles:

- Preserve natural resources and common goods, water, air, soil, climate, biodiversity.
- Optimise and balance the flow of nutrients, mainly nitrogen and phosphorus, in order to reduce the impacts and consumption of resources.
- Minimise the use of sensitive resources and inputs: fertilisers, phytosanitary, products, energy and water.
- Contribute to the local consumption system: the food system and the various productive functions assigned to agriculture.
- Promote ecosystem services such as pollination, biological control and climate regulation.

- Promote specific and genetic biodiversity.

The key practices of agroecology can be listed as follows:

- Associated crops
- Biological control
- Agroforestry
- Eco-grazing
- Legumes
- Plant cover
- Simplified cultivation techniques
- Direct sowing under living plant cover
- Pre-orchards
- Silvopastoralism
- Farmer seeds
- Mixtures meadows
- Organic manure.

Plant production

The analysis of cropping systems has been based on Eurostat and FAOSTAT data [3,6]. The description of crop production is based on a detailed description of 22 main crops, and broader information on 100 other crops is also taken into account. The 22 main crops represent 90% of agricultural land: 8 cereals, 2 oil crops, 3 legumes crops, 2 cash crops, 3 fodder crops, 2 types of meadows and 2 permanent crops. Each crop is described in terms of yield of products (grain, forage etc) and by-products (crops residues), soil carbon storage, inputs (NPK, energy, water, pesticides). The analysis leads to 50% organic farming and 50% soil conservation in 2050. 10% of the farms are managed in agroforestry and 10% in combined crops, i.e. legumes with cereals. In order to compensate for the decrease in fodder crops, crop rotations are extended by greater diversification, including legumes, field vegetables and some perennial grasses.

Animal production

Main components of change are:

- Reduction of overall livestock population due to changed diets
- More grass and less maize-soya ruminants
- Keeping as much permanent grassland as possible under grazing and reallocation arable land to the production of food rather than feed
- Monogastric breeding systems

Feed coverage and international trade

In 2050, the main balances of international trade are maintained overall, with significant changes for some commodities as can be seen in Supplementary Figure 3.

- For cereals, there is a decrease in production due to water scarcity (especially for corn) and change of land-use and practices.
- On the use side, food consumption increases while feed for cattle decreases. As a result, the net export is maintained at the same level in 2050 compared to 2010.
- Milk balance is close to equilibrium, as for meat and sugar.
- Europe becomes a net exporter of vegetables and remains a net importer of fruits.
- Imports of soya cakes drop to nearly zero.

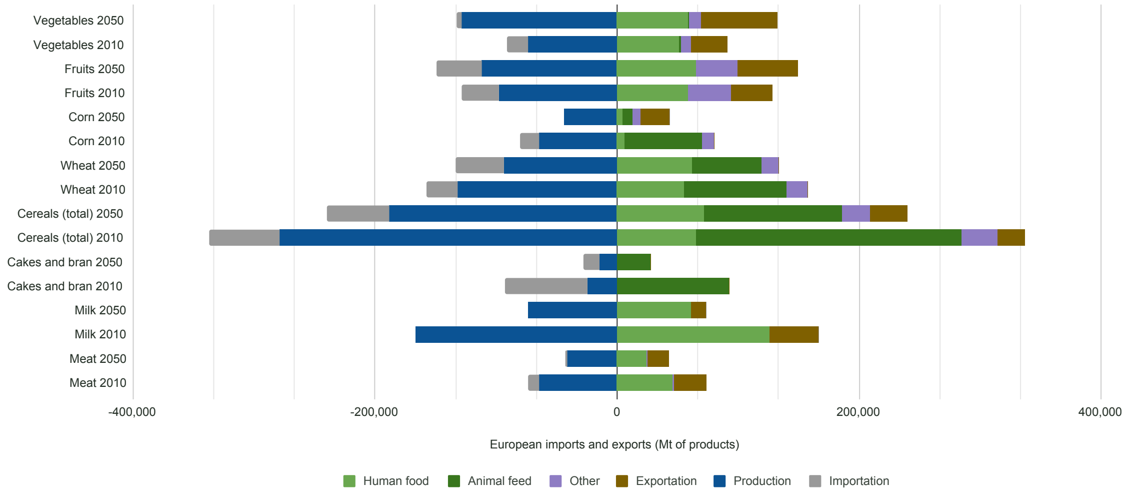

Supplementary Figure 3: Evolution of European imports/exports in Megatonne (Mt) of products. Left side: Resources (production + importation) / Right side: Use (export + domestic demand divided into food, feed and other uses). Exports and imports are expressed as net flows. Single items such as wheat or sugar are either exported or imported. Aggregated categories such as "cereals" and "fruits" may include import flows for some items and exports flows for others. Data and figure are based on Couturier et al. (2022)[2].

Bioenergy potential

Biogas For biogas, the main resources utilised are manure, intermediate coverings, crop residues and grass. Dedicated cultures are completely ruled out by 2050. The different biogas resources, their considered advantages and drawbacks, and relative use in CLEVER are detailed in Supplementary Table 2. The advantages and drawbacks exposed are fundamental, the cost, or difficulty of implementation was considered in the scenario but not in this table. The trajectory was limited to techniques that have already been proven.

Wood and solid bioenergy Solid bioenergy is defined as wood and more generally lignocellulosic materials. Three origins can be distinguished:

- Primary resources extracted directly from ecosystems, the forest, agriculture with hedges, agroforestry, vine shoots and orchard pruning, but also trees in towns, on the roadside or from maintenance of green spaces and gardens.
- Secondary resources, obtained in the primary wood processing industries, sawmills and paper mills.

- Tertiary resources: waste from wood products (materials recovered at the end of the consumption cycle).

Biofuels The biofuels can be distinguished in three generations:

- First generation fuels are fuels produced from dedicated crops (also biogas from excreta). They generally correspond to fuels that began to be produced from oil or grain in the 1990s.
- Second generation fuels are produced from straw.
- Third generation fuels are produced with algae.

Resulting Bioenergy potential in CLEVER

The amount of bioenergy that each country can produce is directly related to its forest area and its agricultural area. There are different national trends following each country's context.

Main summarised results are:

- Slight increase in fuelwood from forest
- The other solid biomass sources increase, this is mainly due to the use of wood by-products and waste and agroforestry installation
- Biogas production increases and represents 2/3 of the overall increase.
- Slight increase for biofuels.

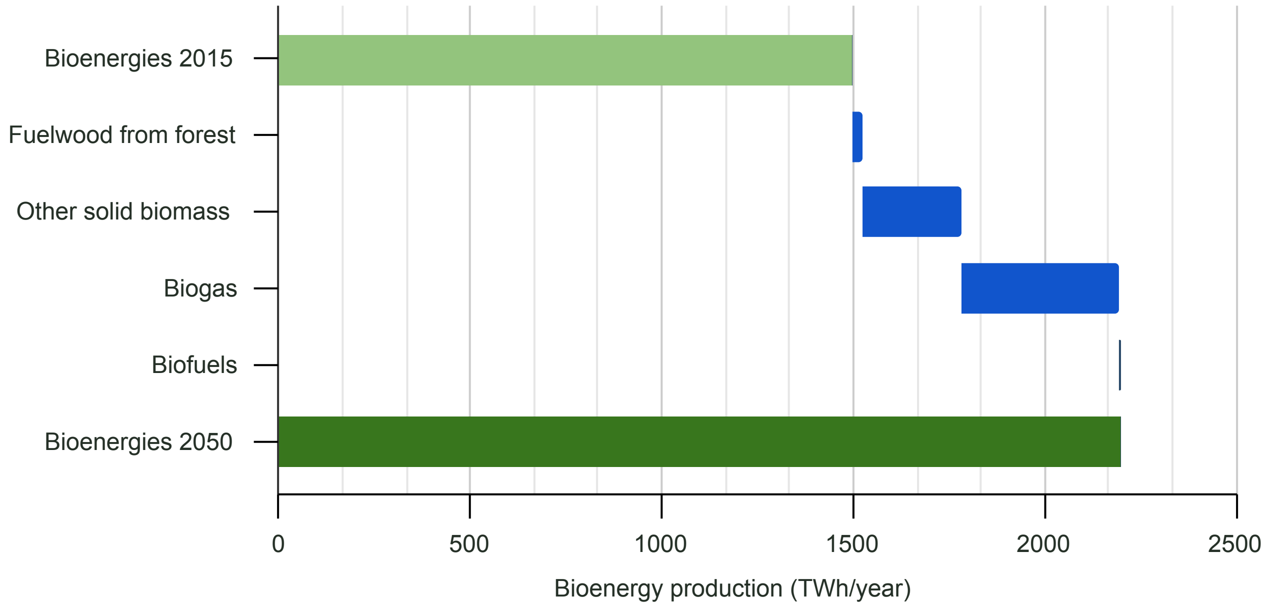

Supplementary Figure 4: Overall change in bioenergy production (EU28, TWh). Data and figure are based on Couturier et al. (2022)[2]. The increase in bioenergies from 2015 to 2050 in the CLEVER scenario is based mainly on biogas and solid biomass and to a minor extent on biofuels.

Supplementary Table 2: Consideration of the use of biogas resources in CLEVER (Collaborative Low Energy Vision for the European Region) based on [2]. Qualitative scale for Use in CLEVER column: ++++: very strong increase compared to today situation; 0: no change to today

Resource	Advantages	Drawbacks	Use in CLEVER
Manure	Improvement of the fertilizing value, no C loss, reduction of GHG emissions during storage	No	+++
Cover crops	Good agroecological practices : N & P recovery, positive impact on erosion, soil organic carbon, biodiversity, pesticide use	Competition for water, reduction of main crop yield, inputs (fuel, N)	++++
Residues (straw)	Low input by-product	Low digestible	++
Dedicated crops	High potential	High competition	0
Grass and fodder	Diversification of outlets in grassland areas as livestock decreases	Competition for feed	++
Municipal and commercial biowaste	Waste treatment	No	+
Wastewater and sludge	Waste treatment	No	+

Supplementary Table 3: Consideration of the use of solid bioenergy in CLEVER (Collaborative Low Energy Vision for the European Region) [2]. Qualitative scale for Use in CLEVER column: +++: strong increase compared to today situation; 0: no change to today

Origin	Resources	Advantages	Drawbacks	Use in CLEVER
Primary	Fuel wood from wood-material oriented forestry	Complementary with construction wood	Risk of nutrient depletion in case of excessive take off	+++
Primary	Fuel wood from dedicated forestry (traditional coppicies)	Traditional method	Possible negative carbon balance	++
Primary	Short rotation coppice	Use of fallow land or water protection areas	Land use	+
Primary	Agroforestry	Good agroecological practices, C storage in soil and wood, favourable to biodiversity	Slight impact on crop yield; no harvest before 10-15 years	+
Primary	Hedges		No	+
Secondary	Sawmills by-products (bark, etc)	Available and low-impact by-products	Competition with other uses (pulp, mulch, compost	+
Secondary	Papermills by-products (black liquor, sludge)			0
Secondary	Agricultural by-products			
Primary	Urban trees, garden and green spaces			+
Tertiary	Wood waste, incl. waste paper, packaging, furniture, carpentry			+
Tertiary	Wood from deconstruction		Emerging practice	+

Supplementary Table 4: Consideration of the use of biofuels in CLEVER (Collaborative Low Energy Vision for the European Region)[2]. Qualitative scale for Use in CLEVER column: +: increase compared to today situation; (+): slight increase compared to today situation

Resource	Advantages	Drawbacks	Use in CLEVER
1st generation biofuels	Easy substitution to fossil fuels	High risk ILUC	+
2nd generation biofuels	Low risk ILUC (under condition)	Technical difficulties	+
3rd generation biofuels	Low risk ILUC (under condition)	Low TRL	(+)

Supplementary Table 5: Comparison of the bioenergy potential applied in the CLEVER (Collaborative Low Energy Vision for the European Region) scenario with other studies. Bioenergies primary production potentials and supply in various scenarios (data are provided in Terawatt hours (TWh) Low Heating Value (LHV) for EU27+UK). Glossary: *Primary production* refers to the definition of EUROSTAT; *Potential* refers to the estimated potential of primary production; *Gross inland consumption* = primary production + imports − exports (here includes maritime and transport, which is a difference to the EUROSTAT definition

	Characterisation of data	Source	EU28 / EU27	Year	Total biofuels	Solid biofuels	Biogas	Liquid biofuels
Domestic primary production	Historical data	EUROSTAT 2023[6]	EU28	2019	1530	1150	195	185
	Potential	CLEVER	EU28	2050	2290	1444	639	208
	Environmental risks' threshold	ESABCC 2023, p. 37 (citing Material Economics 2021)[26,27]	EU27	2050	∼ 2500[1]			
	Potential	JRC (low / high); Castello et al. 2019[28]	EU28	2050	2037 / 5500	>1150 / >3650	173/517 (manure only)	
	Potential	Magnolo et al. 2021[29]	EU27				450-1850 (sequential cropping only)	
	Potential	S2BIOM (European Commission 2020)[30]	EU28		3000 to 6000			
	Scenario results	DE variant / GA variant (ENTSO-E and ENTSOG 2022)[31]	EU27	2050	2064 / 2058	996 / 1169	985 / 789	83 / 100
Gross inland consumption (including international transports)	No bioenergy imports	CLEVER	EU28	2050	2140	1243	622	200
	"Use of bioenergy"	1.5TECH / 1.5LIFE / 1.5LIFELB (European Commission 2018, p. 182-183)[32]	EU28	2050	∼ 2785 ∼ 2260 ∼ 1880			
	140-160TWh of bioenergy imports, mainly solid	REG/ALLBNK (European Commission 2020)[30]	EU27	2050	∼ 2600 ∼ 2900	∼ 1250	∼ 580	∼ 580
		Illustrative scenarios of ESABCC 2023[26], p.54	EU27	2040	1470 to 2861			
	150 to 1000TWh of bioenergies imports[2]	DE variant / GA variant (ENTSO-E and ENTSOG 2022)[31]	EU27	2050	2203 to 2650 / 2210 to 3150	996 / 1169	1124 / 946	83-443 100-944

[1] The Material Economics' study do not integrate the potential of sequential cropping
[2] Depends on the meaning of "Liquids (decarbonised)"

Supplementary Discussion: Planetary boundary aspects in the CLEVER scenario

The energy transition scenario presented in this article has been built to meet the constraints of minimising impacts on one planetary boundary, climate change. However, it has followed a principle of deep sustainability: the choices made in the modelling shouldn't hinder the possibility of reaching a safe operating space for the other processes of the Planetary Boundaries (PB) framework[7].

To measure the fulfilment of this principle, an analysis of the impact of the different modelling choices of this scenario on all PB processes could be undertaken. No global assessment of the impact of a sub-global energy transition scenario on planetary boundaries could be found to guide this work. However, the literature on PB downscaling at the sub-global scale[8,9] could be mobilised. In particular:

- The several publications realising this type of downscaling at the EU level[10–14].
- Two publications that carry out an analysis of the PB impacts of the energy sector in the United States[16] and China[15], based on methods that integrate the PB framework into life cycle assessment methods (PB-LCIA).

Based on the analysis of this literature, three conditions are needed to model the impact of the scenario in this article on the PB process:

- First, the definition of a sharing approach to translate the planetary boundaries to an EU scale boundary (possible approaches are listed in[8]. In this article, an equity approach was chosen by applying a PB share proportional to the population size of the EU compared to the world population. This approach was used to define the EU carbon budget in this paper.
- A sound assessment of the current PB impact of the EU should then be established as a baseline for modelling. This could be based on the methods and results of the EU level PB downscaling described above.
- Finally, the evolution of the impact on each process between now and 2050, given the choices made in the scenario, should be modelled. To be consistent with the methodology of this climate change impact scenario, it should assess both the domestic footprint (perturbation of these processes emitted within EU borders) and the consumption footprint (perturbation caused outside EU borders due to local consumption patterns).

Such an impact assessment of an energy transition scenario would be very useful to guide strategic modelling choices, as well as to advise on policy measures. Indeed, analyses limited to possible energy sector developments in China and the US[15,16] already show that opening up impact assessment to all PBs highlights new challenges that could change production decisions. However, the state of research, methods and data is not yet at a stage where this type of analysis can be fully realised. Instead, qualitative and partly quantitative insights have been developed for each boundary to test the principle of deep sustainability also in comparison to other energy transition pathways and studies. These insights are presented below.

Impact linked to the agriculture and alimentation trajectory

Several planetary boundaries are influenced by the choices made in the construction of the agriculture and food strategy. Such a strategy was developed in the construction of this scenario (see Supplementary Method: Bioenergy potential applied in the CLEVER scenario and Couturier et al. 2022[2]). It allowed the bioenergy potential to be defined in the main text. The detailed impact of this scenario is aligned with a similar agroecological transition scenario at the EU level[17].

Biogeochemical flows

One of the outcomes of this trajectory is a sharp reduction in fertiliser use due to a shift towards agroecological practices. This reduction in inputs is part of a new fertilisation strategy in agroecology, using more cover crops, associated crops, agroforestry and agroecological infrastructure (hedges, grass strips, groves, etc.). This strategy is also linked to the bioenergy production capacity developed in the scenario: small-scale biomass energy production units spread across the landscape are modelled, mainly using biomass produced by cover crops and associated trees (with fertiliser returned to the fields thanks to the digesta). One of the key metrics of this strategy is a calculated 60% reduction in nitrogen fertiliser use in 2050 compared to current levels[2]. This reduction from current levels would be enough to bring the EU's domestic footprint for this cycle back into a safe operating space[13]. The reduction of phosphorus fertiliser use has not been modelled but should follow a similar trend.

Freshwater change

The modelling also includes a drastic reduction in summer irrigation (-80%) by reorganising crop use and avoiding crops that require too much water, such as corn. The level of irrigation in the other seasons is maintained. A positive impact on blue water flows could be deduced from this. With regard to the consumption footprint, a change in diet is also modelled with a reduced consumption of meat (see Supplementary Table 1), which has a great potential to reduce the water footprint[18].

Change in biosphere integrity

The shift in agricultural practices towards agroecology is intended to increase agrobiodiversity. The positive effects of cover crops, hedgerows, agroforestry, etc. on biodiversity are well characterised[5].

Land system change

This planetary boundary concerns the proportion of land cover that is forested[7]. On the domestic footprint side, this scenario models a slight increase in forest area between 2015 and 2050 (see Supplementary Figure 2). On the consumption footprint side - which is the main concern as tropical forests play a major role in the functioning of the Earth system[7] - this scenario assumes a major shift in consumption patterns. This includes a focus on bioenergy production from biomass with no increase in first generation biofuel production to avoid indirect land use change patterns (ILUC). Furthermore, as in the case of freshwater use, the change in diets leads to reduced pressure on land[18]. This is reflected in the scenario by a drastic reduction of imports, especially of feed (see Supplementary Figure 3).

Novel entities

This scenario includes a 70% reduction in pesticide use in the fields by 2050 compared to current levels[2]. This represents a significant reduction in the introduction of one of the families of novel entities introduced by humanity identified in the Planetary Boundaries framework[19].

Other impacts

Atmospheric aerosol loading

Several anthropogenic sources of aerosols in Europe have been identified in the literature[20]: vehicular sources, the industry, fuel/oil combustion, biomass combustion and shipping emissions. This scenario is expected to reduce:

- Vehicular source emissions: thanks to electrification (for car traffic) and a shift toward biogas (for larger vehicles), there is a huge reduction of current level of aerosol emission is expected.

- Emissions from electricity power plants: reduced as a result of the phasing out of coal and oil-based electricity production.
- Industry emissions: the strategy in the industrial sector includes significant electrification (especially in the steel and chemical industries[21]).
- Residential heating emissions: the amount of biomass combustion is divided by almost three by 2050 at EU level and systems (stoves and boilers) are replaced by more efficient and less polluting ones. Other aerosol emitting energy vectors such as coal or oil are phased out.

Novel entities

The sufficiency principle applied in this scenario involves a major shift in the consumption of goods, with attention being paid to the repairability and reusability of goods, as well as to their environmental impact (see the supplementary table on the policies developed to support this work). This will have a positive impact on this indicator, both in terms of reducing the source of new entities (in particular plastics) and in terms of assessing the environmental impact of the production of goods (the safety testing of entities introduced by humanity is the priority underlined by this limit[19]).

However, the new technologies developed in this scenario (e.g. renewable energy, batteries) release technology critical elements (TCEs)[22], including rare earth elements, platinum group elements and scarce metals. These elements have impacts both during their extraction - water pollution, but also deforestation and land degradation due to mining activities - and at the end of their life, with potential pollution associated with their release or recycling. The potential for damage of these elements is one of the motivations for developing a strategy for monitoring critical raw materials, which provides a framework for minimising their consumption. Relative to other scenarios, the CLEVER scenario can partly mitigate the effects due to less required capacity resulting from less demand. Thus, fewer rare and harmful materials are needed to satisfy the creation of renewable technologies. Furthermore, fewer and smaller cars as assumed in the scenario limit battery requirements.

Biosphere integrity

The installation of renewable energy production capacity has a negative impact on biosphere integrity. A full assessment of the literature on this issue is available in[23]. In the case of this scenario, the production of hydroelectric capacity in Europe remains stable, while there is a huge increase in the production of wind and solar energy. This impact is firstly minimised by minimising the need for new electricity generation capacity through the sufficiency principle underlying this scenario. This results in slightly less renewable capacity being developed than projected in the current policy commitments[24] [p.60]. It is then minimised for solar energy by prioritising the development of rooftop solar capacity rather than open space.

Several assumptions in this scenario include a reduction in the need for soil sealing. In particular, a target corridor is set to frame the living space per capita, with a cap of 40m2 per capita (see Figure 3.a in the main text). In addition, a reduction in the need for roads is modelled due to a decrease in road traffic and vehicle size. To translate this ambition, zero net land consumption by 2050 at the latest in the EU countries is one of the policy instruments of this scenario.

Reducing soil sealing should have a positive impact on biodiversity, as there is no possibility to develop ecosystems with significant functions on sealed soils[25].

Supplementary Note: Main Policy Strategies to achieve energy sufficiency in the CLEVER scenario by sector

Policy strategies are the targeted goal of instruments. Example instrument types were categorised by policy instrument type according to the standards of UNFCCC (2000), with the category including plans and strategies not fitting into established categories. For each example, the instrument type is indicated in brackets. The full list of proposed policy instruments is included in the Supplementary Data 2.

The shares of instrument types vary in the sectors agriculture, building, mobility, industry and cross-sectoral but all cover a variety of instrument types as can be seen in Supplementary Figure 5.

Supplementary Table 6: Overview sufficiency policy strategies in the CLEVER (Collaborative Low Energy Vision for the European Region) scenario

Sector	Policy Strategies	Example Instrument (Instrument Type)
Cross-sectoral limits to production	Encourage cultural change towards sufficiency, limits to consumption	Progressive tariff systems for energy and water (economic - pricing)
Agriculture	Reduction of food waste and overconsumption, reduction of live stock	Vegan/ vegetarian dishes / organic food served at catering kitchens (voluntary agreements); Minimum environmental criteria for collective food procurement and preparation for public administration (regulation)
Buildings	Reduce electricity and heat use in buildings, reduce living space, sustainable urban- and settlement development without new soil sealing, efficient use of existing buildings instead of new buildings	19 °C set point in winter in public buildings (regulation) and awareness raising campaigns for consumers (information), limit land take/soil sealing (regulation), support collective living and encourage swapping of households (fiscal and information).
Industry	Exnovation, increase durability of products, increase transparency of environmental impacts, reduce waste, strengthen regional economies	Tax bonus for repairing objects (fiscal - subsidy), prohibit planned obsolescence (regulation), define sufficiency criteria such as size and weight parameters in Ecodesign, labelling and car emissions regulations (regulation), minimum targets for recycled content in new products and set targets for recycling capacity (regulation), integrating consumption reduction targets for materials (for base and critical raw materials) and limiting the growth of strategic raw materials use
Mobility	Improve public transport and multimodality, promotion of active modes, reduce air transport, reduce motorised individual transport, reduce trips (work, personal business, tourism), improve freight infrastructures), promote small and light rail, substitute "accessibility" goals to vehicles	Integrated urban planning, promote 15-minutes city model (fiscal - public expenditures for infrastructure), improved reduce rail and active mobility infrastructure (fiscal - public expenditures for infrastructure), promote carpooling (fiscal - subsidy and infrastructures), promote small and light vehicles (regulation), flight bans where a suitable alternative is available (regulation)

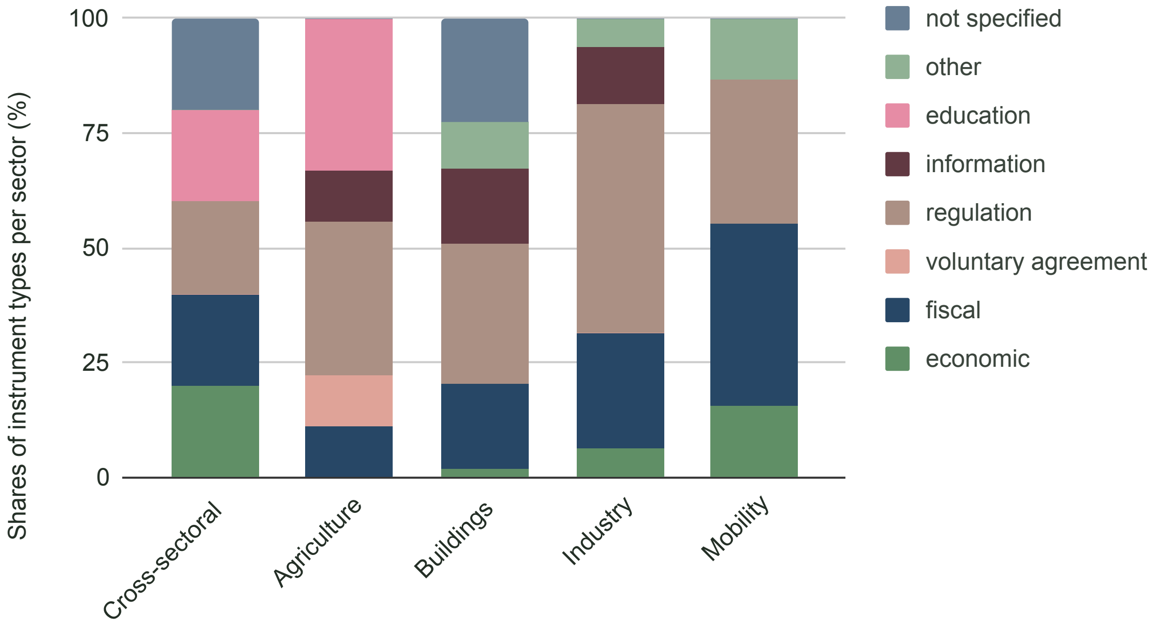

Supplementary Figure 5: Types of instruments for sufficiency applied in the CLEVER (Collaborative Low Energy Vision for the European Region) scenario by sector. For a sufficiency policy mix, all instruments types are used, the distribution varies depending on the sector. Regulatory instruments are important for sufficiency policy in all sectors, but fiscal instruments also play an important role followed by economic, education, information and other instruments.

Supplementary Table 7: Overview countries in the CLEVER (Collaborative Low Energy Vision for the European Region) scenario

Abbreviation	Country
AT	Austria
BE	Belgium
BG	Bulgaria
CH	Switzerland
CY	Cyprus
CZ	Czech Republic
DE	Germany
DK	Denmark
EE	Estonia
EL	Greece
ES	Spain
FI	Finland
FR	France
HR	Croatia
HU	Hungary
IE	Republic of Ireland
IT	Italy
LT	Lithuania
LU	Luxembourg
LV	Latvia
MT	Malta
NL	Netherlands
NO	Norway
PL	Poland
PT	Portugal
RO	Romania
SE	Sweden
SI	Slovenia
SK	Slovakia
UK	United Kingdom
EU27	AT, BE, BG, CY, CZ, DE, DK, EE, EL, ES, FI, FR, HR, HU, IE, IT, LT, LU, LV, MT, NL, PL, PT, RO, SE, SI, SK
EU30	AT, BE, BG, CH, CY, CZ, DE, DK, EE, EL, ES, FI, FR, HR, HU, IE, IT, LT, LU, LV, MT, NL, NO, PL, PT, RO, SE, SI, SK, UK

Supplementary References

1. Couturier, C., Charru, M., Doublet, S. & Pointereau, P. The Afterres scenario 2050. Solagro Association, https://solagro.org/images/imagesCK/files/publications/f94_afterres2050-eng.pdf (2016).

2. Couturier, C., Charru, M., Djelali, M. & Balembois, É. Agriculture, forestry, other land-use changes and bioenergy (AFOLUB) Main assumptions and preliminary trajectory of the CLEVER scenario. https://clever-energy-scenario.eu/wp-content/uploads/2023/03/2303-CLEVER-AFOLUB-note.pdf (2022).

3. FAOSTAT. Food balance sheets 2010-2021. Global, regional and country trends. FAOSTAT Analytical Brief Series No. 72. (2023).

4. FAO and WHO. Sustainable healthy diets – Guiding principles. Rome, https://www.fao.org/3/ca6640en/CA6640EN.pdf (2019).

5. Bezner Kerr, R. *et al.* Agroecology as a transformative approach to tackle climatic, food, and ecosystemic crises. *Current Opinion in Environmental Sustainability* **62**, 101275, https://doi.org/10.1016/j.cosust.2023.101275 (2023).

6. EUROSTAT. Complete energy balances, https://ec.europa.eu/eurostat/databrowser/view/NRG_BAL_C__custom_9275294/default/table?lang=en (2023).

7. Richardson, K. *et al.* Earth beyond six of nine planetary boundaries. *Science advances* **9**, 16, https://doi.org/10.1126/sciadv.adh2458 (2023).

8. Bai, X. *et al.* Translating Earth system boundaries for cities and businesses. *Nature Sustainability* 1–12, https://doi.org/10.1038/s41893-023-01255-w (2024).

9. Chen, X., Li, C., Li, M. & Fang, K. Revisiting the application and methodological extensions of the planetary boundaries for sustainability assessment. *Science of The Total Environment* **788**, 147886, https://doi.org/10.1016/j.scitotenv.2021.147886 (2021).

10. Hoff, H., Nykvist, B. & Carson, M. Living well, within the limits of our planet? Measuring Europe's growing external footprint. Working Paper 2014-05, Stockholm Environment Institute, https://mediamanager.sei.org/documents/Publications/SEI-WP-2014-05-Hoff-EU-Planetary-boundaries.pdf (2014).

11. Häyhä, T., Cornell, S. E., Hoff, H., Lucas, P. & Van Vuuren, D. Operationalizing the concept of a safe operating space at the EU level - first steps and explorations. A report for the European Environment Agency, https://stockholmuniversity.app.box.com/s/hajg8ru0ihvxj8d5topjqp87285c4rj6 (2018).

12. European Environment Agency. & Federal Office for the Environment FOEN. *Is Europe living within the limits of our planet? An assessment of Europe's environmental footprints in relation to planetary boundaries.* Publications Office, LU, https://data.europa.eu/doi/10.2800/890673 (2020).

13. Sala, S., Crenna, E., Secchi, M. & Sanyé-Mengual, E. Environmental sustainability of European production and consumption assessed against planetary boundaries. *Journal of Environmental Management* **269**, 110686, https://doi.org/10.1016/j.jenvman.2020.110686 (2020).

14. Barth, J., Lavorel, C., Miller, C. & Hafele, J. A compass towards 2030 : navigating the EU's economy beyond GDP by applying the Doughnut Economics framework. ZOE Institute for Future-fit Economies, Bonn, https://zoe-institut.de/wp-content/uploads/2023/09/ZOE_Report_Towards-Europe.pdf (2021).

15. Zhang, Y., Wang, Z. & Li, S. Can a new power system help maintain planetary boundaries within a safe operating space? *Energy* **304**, 132030, https://doi.org/10.1016/j.energy.2024.132030 (2024).

16. Algunaibet, I. M. *et al.* Powering sustainable development within planetary boundaries. *Energy & Environmental Science* **12**, 1890–1900, https://doi.org/10.1039/C8EE03423K (2019).

17. Schiavo, M., Le Mouël, C., Poux, X. & Aubert, P.-M. The land use, trade, and global food security impacts of an agroecological transition in the EU. *Frontiers in Sustainable Food Systems* **7**, https://www.frontiersin.org/articles/10.3389/fsufs.2023.1189952 (2023).

18. Aleksandrowicz, L., Green, R., Joy, E. J. M., Smith, P. & Haines, A. The Impacts of Dietary Change on Greenhouse Gas Emissions, Land Use, Water Use, and Health: A Systematic Review. *PLOS ONE* **11**, e0165797, https://doi.org/10.1371/journal.pone.0165797 (2016).

19. Persson, L. *et al.* Outside the Safe Operating Space of the Planetary Boundary for Novel Entities. *Environmental Science & Technology* **56**, 1510–1521, https://doi.org/10.1021/acs.est.1c04158 (2022).

20. Viana, M. *et al.* Source apportionment of particulate matter in Europe: A review of methods and results. *Journal of Aerosol Science* **39**, 827–849, https://doi.org/10.1016/j.jaerosci.2008.05.007 (2008).

21. Toledano, A. *et al.* Establishment of energy consumption convergence corridors to 2050 - Industrial sector. https://clever-energy-scenario.eu/wp-content/uploads/2023/02/2206-Convergence-corridors-Industry.pdf (2022).

22. Bierbaum, R. *et al.* Novel entities and technologies: Environmental benefits and risks. *Environmental Science & Policy* **105**, 134–143, https://doi.org/10.1016/j.envsci.2019.11.002 (2020).

23. Bøe, V., Holden, E. & Linnerud, K. Measuring renewables' impact on biosphere integrity: A review. *Ecological Indicators* **156**, 111135, https://doi.org/10.1016/j.ecolind.2023.111135 (2023).

24. Bourgeois, S. *et al.* Climate neutrality, Energy security and Sustainability: A pathway to bridge the gap through Sufficiency, Efficiency and Renewables. Association négaWatt, https://clever-energy-scenario.eu/wp-content/uploads/2023/10/CLEVER_final-report.pdf (2023).

25. Louwagie, G. *et al.* Impact of land take and soil sealing on biodiversity. *Urban Expansion, Land Cover and Soil Ecosystem Services* (2017).

26. ESABCC. Scientific advice for the determination of an EU-wide 2040 climate target and a greenhouse gas budget for 2030-2050, https://doi.org/10.2800/609405 (2023).

27. Economics, M. EU Biomass use in a Net-zero Economy - a course correction for EU biomass, https://materialeconomics.com/publications/publication/eu-biomass-use (2021).

28. Ruiz Castello, P. *et al.* ENSPRESO - an open data, EU-28 wide, transparent and coherent database of wind, solar and biomass energy potentials. Publisher: European Commission, http://data.jrc.ec.europa.eu/collection/id-00138, https://publications.jrc.ec.europa.eu/repository/handle/JRC116900 (2019).

29. Magnolo, F. *et al.* The Role of Sequential Cropping and Biogasdoneright™ in Enhancing the Sustainability of Agricultural Systems in Europe. *Agronomy* **11**, 2102, https://doi.org/10.3390/agronomy11112102 (2021).

30. Commission, E. IMPACT ASSESSMENT - Stepping up Europe's 2030 climate ambition - Investing in a climate-neutral future for the benefit of our people. SWD, 176 final, Brussels, https://eur-lex.europa.eu/legal-content/EN/TXT/?uri=CELEX%3A52020SC0176 (2020).

31. ENTSO-E and ENTSOG. Ten Year Network Development Plan 2022 Scenario Report. Report: https://2022.entsos-tyndp-scenarios.eu Data File: https://2022.entsos-tyndp-scenarios.eu/wp-content/uploads/2022/04/220228_Updated_Energy_Supply.xlsx (2022).

32. Commission, E. A Clean Planet for all. Communication from the Comission to the European Parliament. COM, 773 final, https://eur-lex.europa.eu/legal-content/EN/TXT/?uri=CELEX:52018DC0773 (2018).
