# Supplementary material for: The key role of sufficiency for low demand-based carbon neutrality and energy security across Europe
Source: Nat Commun. 2024 Oct 19;15:9043. doi: 10.1038/s41467-024-53393-0 (PMC11490562; doi:10.1038/s41467-024-53393-0)
Supplement: Supplementary file 3 — Description of Additional Supplementary Files [file 41467_2024_53393_MOESM3_ESM.pdf]

Description of Additional Supplementary Files

Supplementary Data 1: contains an extract of detailed assumptions / key parameter for each country and sector (one sheet per country). In the sheet „policies“ a table of policy instruments for all sectors considered for the scenario is provided. Furthermore, in the sheet „policies_pivot_table“, the resulting numbers for clustering by sector and instrument type are provided.
